# Patient misidentification and data integrity risks in ultrasound imaging: a case report using multi-framework incident analysis

**DOI:** 10.3389/frhs.2026.1872556

**Published:** 2026-09-03

**Authors:** Benedicta Osei-Donkor, Abdallah Hassoun, Md Shafiqur Rahman Jabin

**Affiliations:** 1 Faculty of Health and Social Care, University of Bradford, Bradford, United Kingdom; 2 Region Gävleborg, Healthcare Common Resources, Gävle, Sweden; 3 Department of Medicine & Optometry, Linnaeus University, Kalmar, Sweden

**Keywords:** diagnostic delay, human factors, interoperability resilience, latent conditions, metadata integrity, sociotechnical system

## Abstract

**Background:**

Digital imaging workflows depend on accurate scheduling, patient identity reconciliation, and reliable metadata continuity to ensure safe image storage and retrieval. Disruptions in these processes may not directly affect image acquisition but can delay access to imaging information and pose significant patient safety risks.

**Objective:**

To examine a recurrent ultrasound workflow failure that compromised downstream image availability and to identify practical strategies for preventing recurrence.

**Methods:**

A retrospectively reported ultrasound incident was examined using a structured case report approach. The event was analyzed using the International Classification for Patient Safety, the Health Information Technology Classification System, the 18-step medical imaging workflow model, a mini-root cause analysis, and a structured risk-control matrix. These complementary frameworks were integrated to classify the incident, localize workflow failures, identify causal mechanisms, and develop preventive and corrective strategies.

**Results:**

The incident was classified as a health information technology–related technical and workflow failure with no confirmed patient harm but clear potential for delayed diagnosis and patient misidentification. The failure originated from missing bookings, modality worklist bypass, and manual patient identity entry, resulting in downstream interoperability failures that delayed image retrieval and interpretation. The mini-root cause analysis identified two interacting causal layers: execution-level workflow deviations and latent system vulnerabilities. Risk-control analysis demonstrated that recurrence was primarily driven by inadequate validation safeguards, weak workflow enforcement, and ineffective exception handling.

**Conclusion:**

This case demonstrates that seemingly minor workflow deviations can evolve into clinically significant patient safety risks despite technically successful image acquisition. The integrated multi-framework analytical approach provides a practical method for identifying latent sociotechnical vulnerabilities, informing targeted risk-control strategies, and strengthening the safety and resilience of digital imaging workflows.

## Introduction

Health information technology (HIT) underpins contemporary medical imaging practice, where safe and effective care depends on the reliable exchange of patient, procedural, and image data across interconnected digital systems (1, 2). In ultrasound imaging, clinical workflow depends heavily on accurate interoperability between booking systems, modality worklists, imaging devices, short-term storage platforms, and long-term archives. Failures within this digital chain may not interrupt image acquisition itself, but can compromise the integrity, accessibility, and traceability of imaging data, creating latent risks to patient safety and clinical decision-making (3, 4).

In digitally mediated imaging environments, clinically significant failures often arise from breakdowns in information flow rather than image acquisition itself. Missing bookings, incorrect patient identifiers, incomplete metadata, and interoperability failures may prevent examinations from being stored, retrieved, or attributed correctly, creating latent risks of delayed diagnosis, patient misidentification, confidentiality breaches, and workflow disruption despite the absence of immediate technical failure (5–7).

These incidents are inherently sociotechnical, arising from interactions among technical systems, clinical workflows, human factors, and organizational processes rather than isolated user or device failures [Carayon et al. (8); Sittig and Singh (6)]. This is particularly relevant in ultrasound, where examinations are often performed in urgent or decentralized settings, increasing reliance on operator judgment and local workflow adaptation, thereby heightening the risk of workflow deviations and metadata-related errors (6, 9, 10).

Structured classification frameworks help characterize such incidents, but classification alone is insufficient to support risk reduction. Frameworks such as the International Classification for Patient Safety (ICPS) (11–14), the Health Information Technology Classification System (HIT-CS) (4, 15, 16), and workflow-based process mapping can describe incident type, technical mechanism, contributing factors, and operational location of failure (2, 17, 18). However, these approaches are primarily descriptive and retrospective, focusing on classifying and understanding events that have already occurred rather than evaluating the adequacy of existing safeguards or identifying prospective system-level interventions to prevent recurrence (4, 19–21).

For this reason, a risk-informed analytical approach is required. Risk-informed analysis extends incident classification by linking identified hazards, contributing conditions, and workflow consequences to existing safeguards, proposed controls, and residual risk. This approach allows incident analysis to move beyond the description of what occurred toward a more practical understanding of why the event remained possible within the system and how similar failures may be prevented in the future (9, 10, 22, 23). In digitally dependent imaging environments, this is particularly important because low-visibility workflow failures may pose clinically meaningful risks through delayed detection, inaccessible information, and compromised data integrity, rather than immediate device malfunction.

Although previous studies have described health information technology–related imaging incidents and interoperability failures, most have focused on incident classification or retrospective event description (17, 24–27). This case extends the existing literature by demonstrating how complementary analytical frameworks can be integrated to classify the incident, localize workflow failures, identify underlying sociotechnical mechanisms, and translate these findings into structured risk-control strategies (28, 29). Beyond describing the incident itself, the study provides a practical analytical approach that may help healthcare organizations investigate similar digital imaging incidents and strengthen workflow resilience.

This case report describes a HIT-related ultrasound workflow incident in which missing bookings, manual identity entry, and workflow bypass resulted in unverified examinations, inaccessible studies, and risk of patient misidentification within the digital imaging chain. Although the available case documentation identified no confirmed patient harm, the incident created observable operational and information-integrity consequences and introduced potential patient safety hazards related to delayed access, incorrect identity attribution, diagnostic error, and confidentiality exposure. This case report analyzes the incident using complementary classification and risk-informed approaches to better understand how the failure emerged, how it propagated across the imaging workflow, and how recurrence may be reduced.

## Methods

### Study design

A qualitative retrospective case report design was used to examine a single medical imaging–related HIT incident involving a workflow failure in ultrasound, a patient identity mismatch, and incomplete interoperability across integrated imaging systems. A case report design was appropriate because the incident represented a context-dependent sociotechnical failure involving interactions among user behavior, software logic, workflow configuration, and digital infrastructure, requiring in-depth analysis of both technical and operational conditions rather than measuring frequency or prevalence (19, 20, 28).

The analytical approach combined deductive and inductive methods to support the structured interpretation of incidents. Deductive analysis was used to classify the incident using predefined patient safety and HIT frameworks, while inductive content analysis was used to identify recurrent operational and organizational themes emerging from the incident narrative and internal investigation records (4, 15, 16, 30). This mixed qualitative approach is consistent with established methods for analyzing complex HIT-related patient safety incidents, particularly where technical and workflow failures are closely interdependent (25, 26, 31). As a descriptive qualitative case report based on a single retrospectively reported incident, the study did not include statistical analyses or hypothesis testing.

### Data source and case selection

The incident analyzed in this study was derived from an anonymized hospital-based patient safety case involving a failure in ultrasound workflow and downstream image accessibility concerns in a Swedish healthcare setting. The case was identified through an internal clinical engineering and imaging systems review following repeated operational disruption affecting ultrasound examination storage, retrieval, and patient identity handling.

The event had undergone routine local technical and workflow investigations as part of an institutional quality and safety review before the retrospective analysis for this study. Documentation available for analysis consisted of deidentified incident material generated during internal review, including the original frontline incident narrative, technical follow-up records, and summary investigation notes. These materials were subsequently examined as secondary quality-improvement data for scholarly analysis.

The case was selected because it represented a recurrent workflow-integrity failure involving both technical and operational contributors, required an urgent local review due to repeated disruptions to the imaging workflow, and provided sufficient technical and contextual detail to support structured qualitative analysis. The case was also considered analytically valuable because it illustrated broader patient safety risks associated with digital interoperability, identity handling, and workflow dependency in ultrasound imaging. The case also supports patient safety learning, technical quality improvement, and education related to medical devices and HIT systems (32).

#### Data collection and case preparation

Data were collected retrospectively from deidentified institutional incident documentation generated during internal technical and workflow review of the event. The primary analytical material consisted of two narrative sources: (1) the incident description, documented at the point of event recognition by frontline clinical personnel, and (2) the summary of investigation, documenting the subsequent internal technical and workflow review undertaken in response to the incident. These materials were used to reconstruct the case chronologically and to compare frontline operational experience with *post hoc* technical interpretation. Box 1 presents the structured case summary used for analysis (33, 34).

BOX 1 **Description of a health information technology–related incident involving patient misidentification and unverified ultrasound examinations due to workflow bypass and interoperability failure**

**Incident description:** Ultrasound examinations performed in the delivery ward were, in several cases, not correctly transferred through the intended digital imaging workflow. Clinical users performing the examinations were often unaware that completed studies were automatically transmitted from the ultrasound system to the storage platform and therefore did not always follow the required workflow steps for correct storage and archiving. As a result, completed examinations were sometimes stored incorrectly, became unverified in the storage system, or could not be retrieved as expected.

A recurring source of error occurred when no formal booking had been created in the appointment system before the examination. In these situations, users sometimes entered patient information manually on the ultrasound console instead of selecting the patient from the scheduled worklist. When this occurred, the system generated a local examination number and wrote it into the patient identity field, resulting in invalid metadata and preventing downstream systems from processing the study correctly. These examinations frequently became stuck in temporary storage and required manual correction before they could be archived.

A further problem occurred when the patient's identity was not verified before image acquisition. In these cases, studies were occasionally stored under an incorrect patient identity, creating a risk of patient misidentification, incorrect attribution of diagnostic findings, and confidentiality breaches. The storage system could not automatically resolve these identity mismatches once the examination had been transmitted.

**Summary of investigation:** The internal investigation identified repeated workflow deviations at both execution and system levels. At the execution level, many examinations were performed without a valid booking in the medical record system, resulting in an invalid accession number and a failure of the automated storage workflow. This was most common during urgent examinations, under workflow pressure, or when users were unaware of the importance of booking and worklist selection. Additional contributing factors included manual entry of incomplete or invalid patient identifiers, expired bookings, use of a different ultrasound device from the originally scheduled one, and failure to verify identity before acquisition.

At the system level, the storage workflow was highly dependent on complete and correct metadata but lacked sufficient safeguards to prevent invalid studies from progressing through the workflow. The ultrasound system did not provide a visible warning when incorrect or incomplete data were entered, and the storage system could not automatically reconcile studies with missing bookings, invalid accession numbers, or incorrect patient identifiers. As a result, unverified studies accumulated in temporary storage and required retrospective manual correction. A software-related anomaly affecting two ultrasound systems was also identified, contributing to repeated workflow failure under specific conditions.

Measures identified in the original hospital investigation: The internal investigation documented initial corrective measures and subsequently proposed preventive and corrective actions. Initial corrective measures included follow-up with users who had submitted incorrectly completed examinations, reinforcement of correct workflow procedures, and targeted communication to improve awareness of booking, worklist use, identity verification, and examination closure. These measures reduced recurrence but did not eliminate the problem.

The hospital investigation subsequently recommended preventive and corrective actions including strengthening user training, restricting workflow bypass, introducing clearer system warnings for incomplete or invalid metadata, and implementing safeguards to prevent studies from progressing without valid booking and identity information. Additional recommendations included creating a dedicated quarantine area for unverified examinations, strengthening monitoring of temporary storage, improving interoperability checks between systems, and adding explicit procurement requirements to ensure future ultrasound systems comply with DICOM workflow and interoperability standards.

Box 1 presents the structured case summary used for analysis, including the findings and control recommendations documented in the original hospital investigation. These pre-existing recommendations were treated as part of the case context and were subsequently examined and mapped against the findings generated through the multi-framework analysis.

Researchers with complementary expertise in health information technology, medical imaging workflows, patient safety, qualitative research, and incident analysis conducted the data extraction and analysis. The research team's multidisciplinary expertise supported consistent interpretation of the incident documentation, workflow reconstruction, and application of the analytical frameworks. Throughout the analysis, the authors discussed their interpretations to enhance analytical consistency and ensure that the findings accurately reflected the available evidence.

To preserve confidentiality and institutional anonymity, the authors removed or replaced all identifiable hospital, system, vendor, and product information with generic functional descriptors before analysis. No patient identifiers were included in the material reviewed. The original case documentation was recorded in Swedish and translated into English for analysis and reporting. A bilingual linguistic specialist familiar with technical terminology in Swedish and English supported the translation. The translated material was subsequently reviewed by the principal investigator against the original source text to preserve linguistic consistency, contextual meaning, and technical interpretive fidelity (35).

#### Clarification of technical identifiers and workflow information

Because the incident involved the interaction of patient identification, booking, examination, and downstream imaging systems, these types of information were distinguished throughout the analysis. Patient ID refers to the identifier used to identify the patient, whereas the accession number links an imaging examination to its corresponding booking or imaging request. Modality worklist information provides the patient and examination information available to the imaging device for selection and workflow initiation. A local examination number is an identifier generated by the imaging system for an individual examination and is distinct from the Patient ID and accession number. Study identification refers to the information used by downstream imaging systems to identify and manage the completed imaging study. These terms were therefore treated as distinct analytical concepts when interpreting the incident and its interoperability failures. No actual patient, accession, examination, or other identifying values were retained or reported.

### Analytical framework

The incident was analyzed using a staged, multi-framework approach combining patient safety classification, HIT classification, workflow localization, and risk-informed analysis. This approach was used to improve methodological transparency and support triangulation across complementary analytical lenses (4, 14).

The analytical frameworks were selected because they provide complementary but distinct perspectives on the incident. HIT-CS was used to characterize health information technology–related technical failures, information-processing problems, and system functionality issues specific to digitally mediated healthcare (4, 28). ICPS was selected to classify patient safety contributing factors, incident characteristics, and patient and organizational outcomes using an internationally recognized patient safety framework (11). The validated 18-step medical imaging workflow model was used to localize where the failure originated, propagated, and became operationally apparent within the imaging process (1). Finally, the risk-control matrix was used to map identified hazards and contributing factors against documented existing safeguards and the control measures identified through the case investigation, while organising these measures into preventive and corrective control categories (36). Together, these complementary frameworks enabled technical classification, patient-safety interpretation, workflow localization, causal understanding, and risk-informed recommendations, thereby addressing distinct yet interrelated dimensions of the incident.

Alternative systems-based frameworks, including the Systems Engineering Initiative for Patient Safety (SEIPS) (8, 37, 38), the Human Factors Analysis and Classification System (HFACS) (39–41), AcciMap (42, 43), and the Functional Resonance Analysis Method (FRAM) (44), were considered during study design. These approaches are well suited to examining broader organizational, human factors, and system interactions. However, this study primarily aimed to characterize a health information technology–related imaging incident, localize the failure within the digital imaging workflow, and develop practical risk-control recommendations. Consequently, frameworks specifically designed for HIT incident classification, patient safety classification, workflow localization, and risk management were considered more appropriate for addressing the study objectives.

To ensure clarity and consistency, terminology was applied consistently throughout the manuscript according to the context of the incident description, the analytical frameworks used, and established medical imaging informatics terminology. Terms referring to workflow processes, metadata, patient identity, and imaging records were used deliberately according to their intended technical meaning within the analysis.

First, inductive content analysis was applied to the incident narrative and investigation summary to identify recurrent operational themes, workflow deviations, and contributing conditions. This allowed emergent patterns to be identified directly from the case narrative before formal classification (30).

Second, deductive classification was performed using the Health Information Technology Classification System (HIT-CS) to categorize the incident according to technical domain, failure type, and system function. HIT-CS was used to identify user-, software-, and machine-related dimensions of failure, including incorrect data entry, absent data entry, delayed retrieval, incorrect output, and software functionality issues (4, 45).

Third, the incident was analyzed using the International Classification for Patient Safety (ICPS) to classify contributing factors and outcomes from a patient safety perspective. ICPS was used to identify communication, staff, documentation, system, and patient-related contributors, as well as organizational and patient-level outcomes associated with the event (14).

Fourth, workflow localization was performed using the validated 18-step medical imaging workflow model described by Jabin et al. (2), enabling localization of the incident across the imaging process and identification of where the failure emerged, propagated, and became operationally visible (2).

#### Mini-root cause analysis

A focused mini root cause analysis (RCA) was undertaken to support causal interpretation beyond descriptive incident classification and to further examine why the incident remained possible within the existing workflow. The purpose of this step was not to perform a full, formal organizational RCA, but to conduct a structured secondary analysis of the existing incident documentation to identify immediate operational failures, latent system vulnerabilities, and recurrent enabling conditions (9, 10). This approach was considered appropriate because the available data were derived from an existing anonymized incident reporting system and internal technical investigation records, rather than from prospective interviews, observations, or primary data collection.

The mini-Root Cause Analysis (RCA) was therefore conducted as a document-based analytical exercise using only pre-existing, de-identified incident material available in the reporting system. No additional data were collected from patients, staff, or clinical records, and no direct observation, intervention, or participant contact was undertaken. The analysis was limited to reinterpreting the incident narrative, the internal investigation summary, and the technical follow-up notes already recorded in the reporting repository. As such, the mini-RCA served as an analytical extension of the case review rather than a separate empirical data-collection activity. This approach reduced ethical sensitivity and ensured that causal interpretation remained confined to anonymized quality-improvement material already available for secondary analysis (46, 47).

The mini-RCA followed a structured analytical procedure. First, the incident description, investigation summary, and technical follow-up documentation were reviewed to identify workflow deviations, technical failures, and organizational conditions associated with the event. Second, these findings were examined iteratively using Reason's concepts of active failures and latent conditions together with sociotechnical systems thinking to distinguish execution-level failures from system-level vulnerabilities (6, 9, 29). Third, the emerging interpretations were compared with findings from HIT-CS classification, ICPS classification, workflow localization, and the risk-control matrix to assess consistency across the analytical frameworks. Throughout the analytical process, the authors discussed interpretations to enhance analytical consistency and ensure the conclusions remained grounded in the available incident documentation.

The mini-RCA was not intended to constitute a formal organizational Root Cause Analysis (RCA) based on established methodologies such as the London Protocol (48). Rather, it was undertaken as a structured, document-based causal interpretation informed by Reason's concepts of active failures and latent conditions and supported by sociotechnical systems thinking (6, 9) This approach was considered appropriate because the available evidence consisted exclusively of retrospective, anonymized incident documentation and internal investigation records, which did not permit the comprehensive data collection, stakeholder interviews, multidisciplinary investigation, or systems redesign processes typically associated with formal RCA methodologies (48). The purpose of the mini-RCA was therefore to provide a transparent and theoretically informed interpretation of the causal mechanisms underlying the incident and to complement the descriptive findings generated through incident classification, workflow localization, and risk-control analysis.

The mini-RCA was conducted to identify the causal pathway underlying repeated workflow failure and to distinguish between proximal operational breakdowns and deeper systemic contributors. Specifically, the analysis examined how workflow deviations, local workarounds, software behavior, metadata dependencies, and organizational routines interacted to enable repeated failures in the digital imaging workflow. The RCA, therefore, aimed to clarify not only what failed but also why the same failure could recur despite partial corrective action.

To support this interpretation, the RCA was structured at two analytical levels. The first level, execution-level analysis, focused on active failures that occur during routine clinical operations. This included missed bookings, failure to use the modality worklist, manual entry of identities, incomplete identity verification, and workflow bypass during urgent or administratively complex examinations. These were treated as proximal failures because they directly preceded the incident and were observable within the routine execution of the imaging workflow.

The second level, system-level analysis, focused on latent conditions embedded in the broader sociotechnical environment. This included software behavior, metadata dependencies, lack of visible system warnings, interoperability limitations, weak workflow enforcement, and the storage system's inability to reconcile invalid or incomplete studies. These factors were treated as latent system conditions because they did not directly initiate the incident but created conditions in which active failures could recur without interruption or early detection.

This two-level analytical structure was used to distinguish active failures from latent conditions, consistent with systems-based models of patient safety and sociotechnical risk analysis (6, 9, 10). In this framework, active failures were understood as the immediate actions or omissions occurring at the point of care, whereas latent conditions were understood as structural weaknesses in system design, workflow configuration, and organizational control that allowed these failures to propagate into repeated operational disruption.

The mini-RCA was conducted iteratively and interpretively by reviewing the incident description, the investigation summary, and the identified workflow deviations, alongside findings from ICPS, HIT-CS, and workflow localization. Emerging causal themes were compared across these analytical stages to identify points of convergence between observed workflow failure, technical system behavior, and organizational vulnerability. The RCA therefore functioned as an interpretive bridge between descriptive classification and prospective risk-control analysis, supporting causal explanation and informing the design of preventive and corrective strategies.

#### Risk–control matrix

Following the workflow localization, a structured mini-root cause analysis was undertaken to interpret the interaction between execution-level failures and latent system conditions. Finally, a structured risk–control matrix translated the classification findings into practical risk-management terms. Finally, a structured risk–control matrix translated the classification findings into practical risk-management terms. The matrix linked identified hazards, contributing causes, workflow consequences, existing safeguards, proposed preventive and corrective controls, and residual risk considerations. Initial risk levels were documented from the case analysis, whereas residual risk was not formally recalculated after the proposed controls because these controls had not been implemented or evaluated within the case. The matrix therefore identified control gaps and required safeguards rather than demonstrating a quantified reduction in residual risk. The purpose of this step was not to reclassify the incident, but to evaluate the adequacy of existing controls and identify where preventive system-level safeguards were required (9, 23).

### Analytical rigour

Analytical rigor was supported by methodological triangulation, the structured use of established classification systems, and iterative comparisons across analytical stages. Triangulation was achieved by examining the same incident through multiple complementary frameworks and comparing areas of convergence across patient safety, technical, workflow, and risk domains (49, 50). Although the analytical frameworks addressed some overlapping aspects of the incident, they were intentionally selected because each contributed a distinct analytical perspective. HIT-CS focused on characterizing health information technology–related technical failures and information-processing issues (4, 28), whereas ICPS classified patient safety contributing factors, incident characteristics, and patient and organizational outcomes (14). The 18-step medical imaging workflow model localized where the incident originated, propagated, and became operationally apparent within the imaging process (2). The mini-root cause analysis provided a systems-oriented interpretation of the underlying causal mechanisms (6, 9), while the risk-control matrix translated these findings into practical preventive and corrective strategies based on risk management principles (36). Consequently, areas of conceptual overlap were used to corroborate findings across frameworks, whereas their distinct analytical functions provided complementary insights into the technical, workflow, patient safety, causal, and risk management dimensions of the incident.

Interpretive consistency was strengthened by comparing frontline incident narratives with findings from internal technical investigations and by validating translated case content against the original source text. The staged analytical design improved transparency, strengthened interpretive validity, and reduced reliance on single-framework inference when analyzing a complex HIT-related imaging incident.

## Results

### Overview of incident classification

The incident was analyzed across four complementary analytical layers: incident classification, workflow localization, root cause interpretation, and risk-informed control mapping. Across these analyses, the event was characterized as a HIT-related interoperability failure involving patient identity handling, workflow bypass, and metadata dependency. Ultrasound examinations were completed successfully, but failures in booking, structured workflow use, and metadata validation disrupted downstream storage and retrieval, resulting in unverified examinations and delayed or difficult access to imaging information.

### HIT-CS classification

Using the HIT-CS, the incident was classified as a combined use-related, software-related, and machine-related HIT event (Table 1). The primary issue was use-related and involved incorrect, absent, or delayed data entry and retrieval, including manual patient identification, failure to use the modality worklist, incomplete bookings, and delayed study retrieval from downstream systems. These workflow deviations represented the principal mechanism underlying the incident.

**Table 1:** Classification of the incident using the health information technology classification system (HIT-CS).

**HIT-CS domain**	**Category**	**Subtype**	**Application to this incident**
Use-related issue	Information input/output	Wrong data entry or retrieval	Manual patient entry, incorrect identity assignment, and invalid metadata
Use-related issue	Information input/output	No data entry or retrieval	Missing booking, no valid accession-linked workflow information, no valid worklist linkage
Use-related issue	Information input/output	Delayed or partial data entry or retrieval	Delayed archival, incomplete transfer, retrospective correction required
Software-related issue	Software functionality	Silent workflow failure	No warning for invalid metadata or failed storage conditions
Software-related issue	System logic	Metadata dependency	Workflow dependent on complete accession and identity data
Machine-related issue	Device/system output	Wrong output	Incorrect generation of local examination identifiers
Machine-related issue	Device/system behavior	Software anomaly	Recurrent processing failure in two ultrasound systems

A secondary software-related issue involved system functionality. The storage workflow depended on valid metadata and accession logic, yet the software failed to provide visible warnings when incomplete or incorrect data were entered, allowing examinations to progress unnoticed until they became unverified in storage.

A machine-related issue was also identified. An internal investigation suggested a software anomaly affecting two ultrasound systems under specific operating conditions. Although not the primary cause, this behavior likely contributed to recurrence and increased system vulnerability.

### ICPS classification

Using the ICPS, the incident was classified as a documentation, communication, and patient identification event with both patient-level and organizational consequences. The principal contributing factors included staff, communication, documentation, and system factors, with additional contributions from workload and patient identity complexity.

From an ICPS perspective, the incident represented a patient safety–relevant event involving incomplete information transfer, delayed documentation, identity mismatch, and unsafe workflow adaptation. The documented effects were primarily operational and information-related, including affected studies becoming unverified or difficult to retrieve and requiring manual reconciliation. The available case documentation identified no confirmed patient harm. However, the observed workflow and identity failures created potential patient safety hazards, including wrong-patient attribution, delayed clinical decision-making, diagnostic error, and confidentiality exposure if such failures were not detected and corrected. Organizational implications included increased documentation burden, manual recovery effort, service disruption, and medical-record traceability concerns. Table 2 presents the revised ICPS classification.

**Table 2:** ICPS classification of contributing factors and outcomes.

**ICPS domain**	**Sub-category**	**Application to this incident**
Contributing factor	Communication factor	Incomplete data transfer between users and systems
Contributing factor	Staff factor	Limited awareness of workflow requirements; failure to verify identity
Contributing factor	Workforce factor	Time pressure during urgent examinations
Contributing factor	Documentation factor	Missing booking; delayed or incomplete documentation
Contributing factor	System factor	Lack of warning, poor workflow enforcement, and metadata dependency
Contributing factor	Patient factor	Protected identity; absent or non-standard identity information
Observed operational consequence	Information access/retrieval	Delayed or difficult retrieval of affected studies; delayed workflow resolution
Potential patient safety hazard	Wrong-patient attribution	Risk of incorrect attribution of findings if identity errors were not detected and corrected
Potential patient safety hazard	Diagnostic delay/error	Potential for delayed clinical decision-making or diagnostic error if affected studies remained inaccessible or were incorrectly attributed
Potential information-governance hazard	Confidentiality	Potential exposure of patient information if examination data were associated with an incorrect identity
Organisational consequence	Increased documentation/recovery workload	Manual reconciliation and retrospective correction required
Organisational consequence	Service disruption	Delayed availability of affected studies within the archive
Organisational consequence	Legal/traceability implication	Medical-record integrity and traceability concerns

### Workflow localization using the 18-step medical imaging process

Applying the 18-step medical imaging workflow model showed that the incident originated during the pre-acquisition phase and progressed through subsequent storage and retrieval processes. The primary workflow disruption occurred at Step 3 (Patient booking and scheduling), where no valid accession-linked workflow information was available because no valid booking existed. This failure extended to Step 6 (Pre-examination preparation), where the modality worklist was bypassed or incomplete, and Step 7 (Image acquisition), where patient identifiers were entered manually or incorrectly. Consequently, the examinations could not be correctly validated or archived at Step 8 (Image work-up and transfer).

The operational consequences became apparent at Step 10 (Study selection and retrieval) and Step 12 (Image review and interpretation), where examinations were delayed, inaccessible, or linked to incorrect patient records. Table 3 presents the incident's location within the 18-step medical imaging workflow.

**Table 3:** Localization of the incident using the 18-step medical imaging workflow model.

**Workflow step**	**Workflow stage**	**Relevance to the incident**	**Operational effect**
**Step 3**	Patient booking and scheduling	Primary upstream failure	The missing booking prevented availability of valid accession-linked workflow information
**Step 6**	Pre-examination preparation	Workflow deviation	Worklist bypassed or incomplete
**Step 7**	Image acquisition	Identity handling failure	Manual or incorrect patient identity entry
**Step 8**	Image work-up and transfer	Propagation point	Study transferred with invalid metadata and became unverified
**Step 10**	Study selection and retrieval	Operational detection	Study delayed, inaccessible, or difficult to locate
**Step 12**	Image review and interpretation	Potential clinical consequence	Delayed review and risk of incorrect attribution

**Figure 1:**
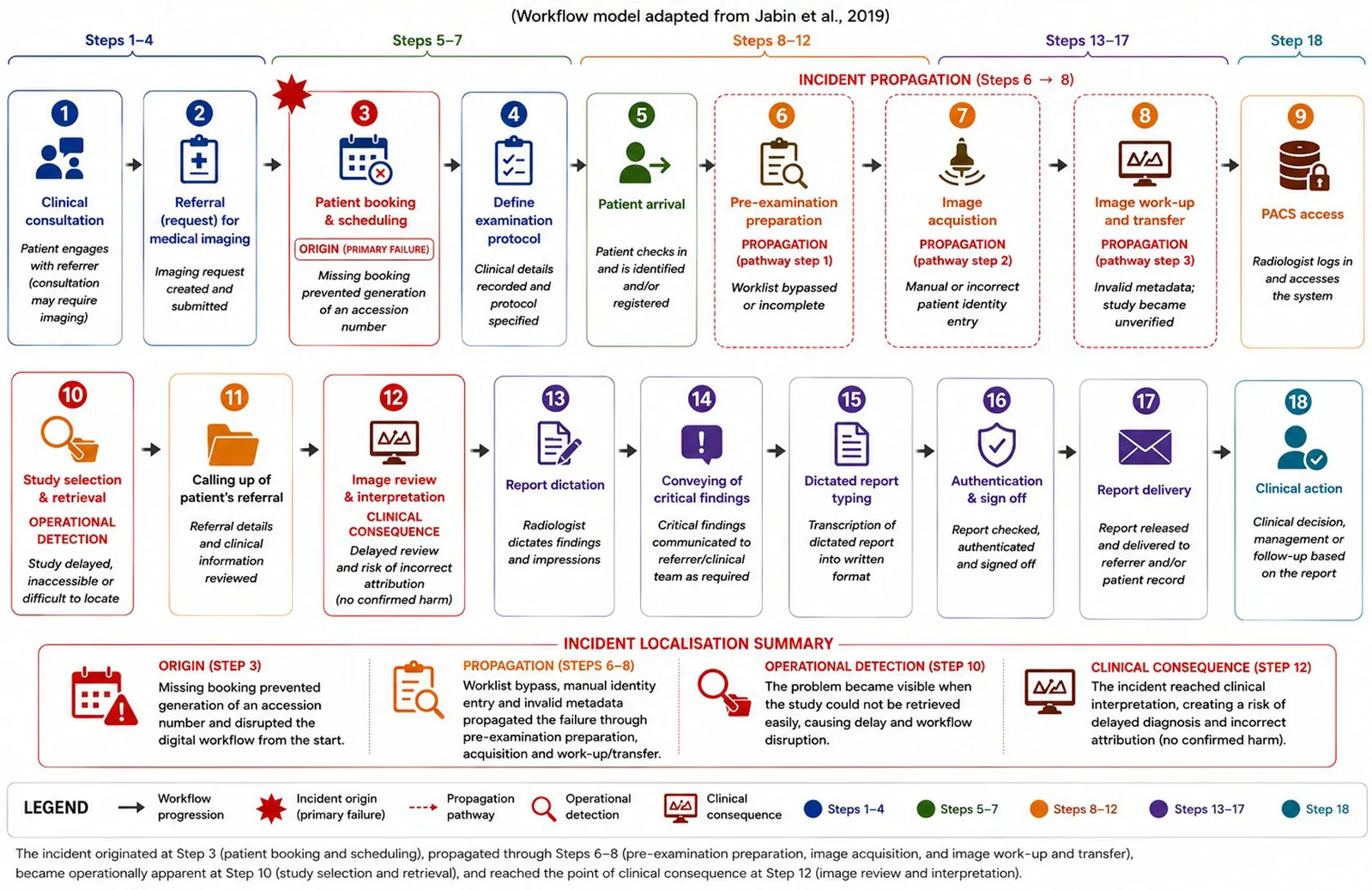
The 18-step medical imaging workflow with localization of the incident.

Figure 1 illustrates the incident localization within the 18-step medical imaging workflow.

The workflow stages are based on the 18-step medical imaging process described by Jabin et al. (2019). The incident originated at Step 3 (patient booking and scheduling), propagated through Steps 6–8 (pre-examination preparation, image acquisition, and image work-up and transfer), became operationally apparent at Step 10 (study selection and retrieval), and reached the point of clinical consequence at Step 12 (image review and interpretation). Incident-localized stages are distinguished from non-localized workflow stages.

### Mini-RCA analysis

The mini-root cause analysis identified two interacting causal layers underlying the incident: execution-level failures and system-level vulnerabilities. Rather than resulting from a single user error or an isolated software malfunction, the incident arose from the interaction between active workflow deviations and latent system weaknesses.

At the execution level, repeated workflow deviations initiated the incident. In the absence of a valid booking, no valid accession-linked workflow information was available, preventing the study from being correctly linked to the downstream storage workflow. Such deviations occurred most frequently during urgent examinations or when patients required non-standard administrative handling, resulting in local workarounds that bypassed established digital workflow requirements. Limited user understanding of the relationship between booking, worklist population, metadata generation, and downstream image storage further contributed to these failures, allowing examinations to enter the digital workflow with incomplete or invalid metadata.

At the system level, latent weaknesses in workflow design, interoperability, and system safeguards allowed these execution-level failures to propagate. The workflow depended on complete, valid metadata, yet inadequate validation controls allowed incomplete bookings, invalid identifiers, and missing accession numbers to progress without interruption. In addition, neither the ultrasound system nor the downstream storage system provided effective warnings or feedback when workflow prerequisites were unmet, or studies remained unverified, thereby delaying error detection and recovery.

The analysis also identified weak workflow enforcement as an organizational contributor. Although valid booking, modality worklist use, and patient identity verification were fundamental workflow requirements, examinations could still proceed when these prerequisites were not fulfilled, increasing reliance on user vigilance, local knowledge, and retrospective correction.

**Figure 2:**
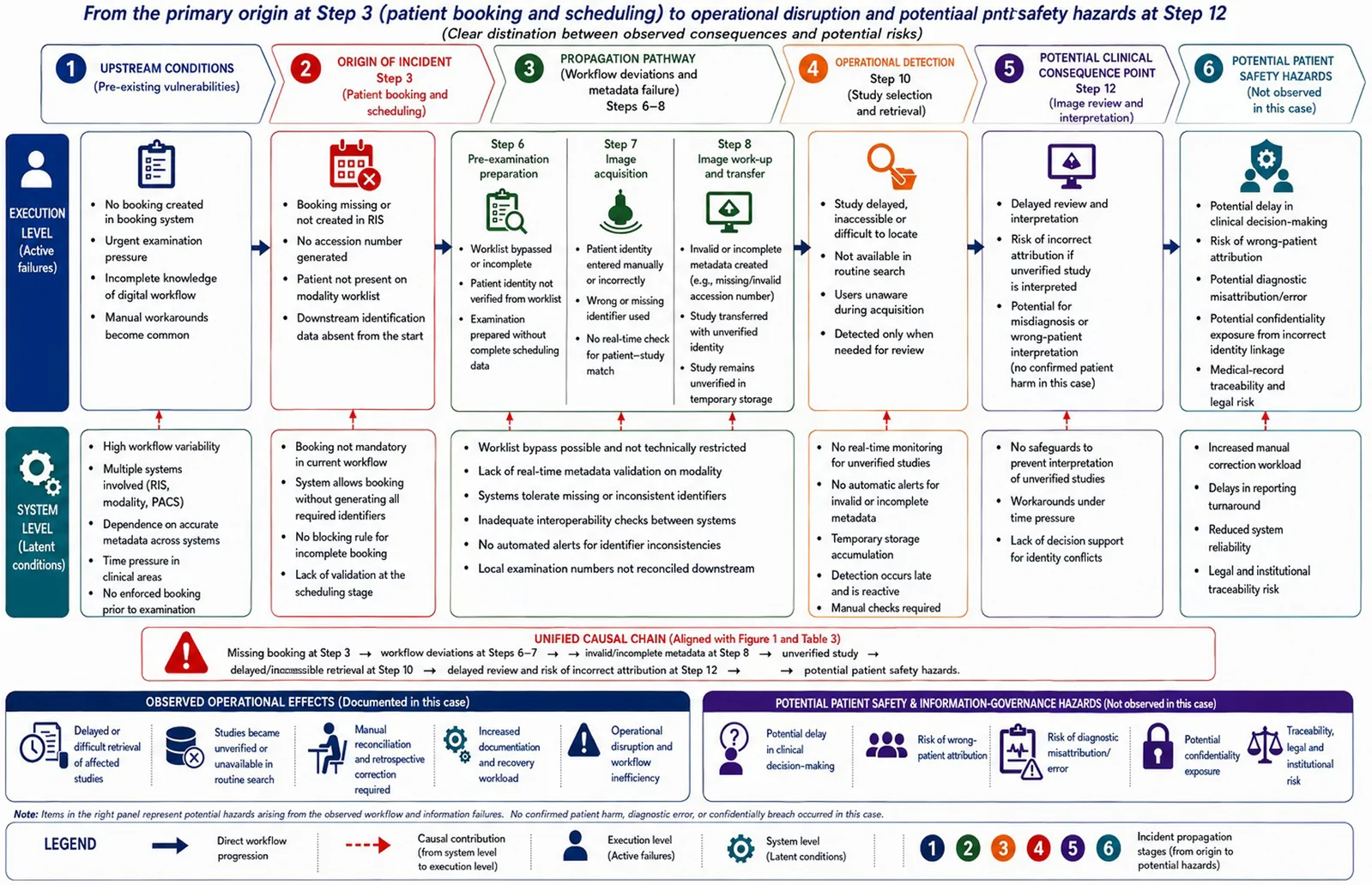
Incident propagation pathway from workflow deviation to operational disruption and potential patient safety hazards.

The analysis, therefore, identified interacting execution-level and system-level conditions that allowed the workflow failure to recur and propagate downstream. Figure 2 illustrates the resulting incident pathway.

### Cross-framework synthesis

Across the analytical frameworks, each method contributed a distinct perspective: HIT-CS characterized the technical and information-processing failures; ICPS identified patient-safety and organizational implications; workflow localization identified where the failure originated, propagated, and became operationally visible; and the mini-root cause analysis examined the interaction between execution-level deviations and system-level conditions. The risk-control matrix then mapped these findings against the documented hospital-investigation recommendations and existing safeguards. Together, the analyses provided a structured account of the incident across technical, workflow, causal, patient-safety, and risk-control dimensions. Figure 2 illustrates the incident propagation pathway.

Figure 2 illustrates the progression of the observed workflow failure from the upstream booking and workflow deviations through invalid metadata, incomplete validation, unverified storage, and delayed or difficult retrieval. The downstream clinical and patient-safety elements represent potential hazards arising from these failures rather than confirmed patient harm or observed diagnostic error. The figure therefore distinguishes documented operational effects from prospective patient safety and information-governance risks.

### Risk–control matrix analysis

To translate the analytical findings into practical risk management strategies, the incident was further evaluated using a structured risk-control matrix. The matrix links workflow hazards to contributing causes, existing safeguards, preventive and corrective controls, and residual risk to identify system-level interventions to reduce incident recurrence.

The risk-control matrix identified high-priority failure modes associated with patient booking, modality worklist use, and patient identity handling. Initial risk levels ranged from medium to very high, with the highest rating associated with wrong-patient selection. Existing safeguards were predominantly reactive and dependent on manual correction, reconciliation, or incident reporting. Priority control strategies focused on booking validation, mandatory worklist use, identity validation, and structured exception handling. Because the proposed controls were not implemented or prospectively evaluated within the case, their effect on residual risk was not formally calculated.

To improve readability, Table 4 summarizes the principal risk-control findings, while Supplementary Table S1 provides the full risk-control matrix, including contributing causes, potential consequences, existing safeguards, proposed preventive and corrective controls, and residual-risk assessment. Table 4 therefore highlights the priority failure modes, initial risk levels, existing safeguards, and principal control strategies identified through the integrated analysis.

**Table 4:** Summary of priority risks and control strategies identified through the risk-control analysis.

**Process step**	**Priority failure mode**	**Initial risk**	**Key existing safeguard**	**Priority control strategy**
Appointment booking	No booking created	High	Post-event booking creation and manual MTA matching	Mandatory booking validation before examination; structured emergency booking pathway
Appointment booking	Incorrect examination type selected	Medium	Manual recoding and reconciliation	Standardized examination codes and restricted free-text options
Modality worklist	Worklist not used	High	Manual correction where identifiable	Mandatory worklist use and system enforcement
Patient identity handling	Manual identity entry	High	Manual review/correction and examination quarantine where applicable	Restrict manual identity entry and implement identity validation
Patient identity handling	Wrong patient selected	Very high	Incident reporting and repeat examination where necessary	Two-step identity verification and confirmation prompts

The full matrix provides the underlying causal, control, and residual-risk details supporting this summary; residual risk was not formally calculated because the proposed controls were not implemented or prospectively evaluated within the case (Supplementary Table S1. Full risk-control matrix for the ultrasound imaging incident).

## Discussion

### Principal findings

This case report examined a recurrent HIT-related interoperability failure in ultrasound imaging in which missing bookings, workflow bypass, and manual identity entry resulted in unverified examinations, delayed image availability, and an increased risk of patient misidentification. Although ultrasound examinations were completed successfully, failures in booking logic, identity management, and metadata validation disrupted downstream storage and retrieval, creating latent risks to the availability, traceability, and correct attribution of imaging information.

These findings highlight that maintaining digital workflow integrity is as important as successful image acquisition for ensuring patient safety. Reliable booking processes, patient identity management, and metadata validation are fundamental prerequisites for ensuring that imaging information remains accessible, traceable, and clinically usable throughout the care pathway. Consequently, imaging safety should be considered within the broader context of sociotechnical system performance rather than being limited to equipment functionality alone (6, 8, 16, 51). Similar HIT-related incidents have demonstrated that disruptions to information flow, rather than hardware failure, may compromise clinical decision-making through delayed access to imaging information, incomplete records, and workflow disruption (24, 27, 52).

A second important finding is that the incident was not attributable to isolated user error but to the interaction between active workflow deviations and latent system vulnerabilities. Missing bookings, modality worklist bypass, and manual identity entry were the immediate operational failures, while inadequate safeguards, weak workflow enforcement, metadata dependencies, and limited interoperability controls enabled their recurrence. This interpretation is consistent with systems-based patient safety theory, which recognizes that incidents emerge through the interaction of active failures and latent organizational or technical conditions rather than isolated individual errors (8, 9).

Importantly, the patient safety implications identified in this analysis should not be interpreted as confirmed patient harm. The available case documentation did not identify a confirmed adverse clinical outcome, diagnostic error, or confidentiality breach. Rather, the observed workflow and information-integrity failures created plausible pathways through which such harm could occur if similar failures remained undetected or were not corrected. Accordingly, the analysis distinguishes documented operational consequences from potential patient safety and information-governance hazards.

### Interpretation through patient safety and sociotechnical perspectives

The integrated analysis supports a sociotechnical interpretation in which users, digital systems, workflow constraints, and organizational practices interacted to produce the observed failure pathway. The temporal separation between failure origin and detection was particularly important: the workflow disruption began during booking and preparation, propagated through acquisition and transfer, and became operationally visible only during retrieval and interpretation (53–55).

This temporal separation between the origin of failure and its detection is clinically important. In digitally mediated imaging environments, data integrity failures often remain hidden until the information is needed for clinical use. Prior studies have shown that delayed detection is a defining feature of HIT-related patient safety incidents, particularly where system failures affect information availability rather than immediate technical function (6, 7, 56). In the present case, examinations were completed successfully and appeared operationally normal at the point of care, but the digital workflow had already failed. This explains why apparently minor workflow deviations, such as bypassing a booking or manually entering identity information, can have disproportionately large downstream consequences.

The case also reinforces the value of workflow-based incident localization. By identifying Step 3 as the primary point of failure, Steps 6–8 as the propagation pathway, Step 10 as the point of operational detection, and Step 12 as the point of clinical consequence, the analysis demonstrated how workflow mapping can localize not only where an incident occurred, but where intervention is most likely to be effective. This supports prior work showing that workflow-based analysis improves the practical interpretability of imaging-related HIT incidents and strengthens targeting of corrective interventions (1, 2).

### Preventive and corrective strategies

The control analysis identified the need for coordinated technical, workflow, and administrative safeguards rather than reliance on user vigilance alone. Several controls in the risk-control matrix had already been recommended in the original hospital investigation; this analysis mapped them to the identified workflow, causal, and risk domains.

At the point of examination, mandatory identity verification and restriction of manual identity entry are also critical. Manual entry of patient identity should be limited to exceptional circumstances and supported by explicit validation rules, visible warnings, and structured exception pathways. This is particularly important in urgent examinations and in patients requiring alternative administrative handling, where local workarounds are most likely to occur.

At the system level, defensive safeguards should be strengthened to prevent silent failure. Systems should provide visible alerts when bookings are absent, accession numbers are missing, identity fields are invalid, or studies fail downstream validation. Studies that cannot be safely reconciled should be automatically diverted to a controlled quarantine pathway rather than remaining silently in temporary storage. These controls align with established safety principles in digital systems, where forcing functions, hard stops, and automated validation are more effective than reliance on user memory or vigilance alone (6, 8, 38).

At the organizational level, interoperability requirements should be treated as explicit patient safety requirements rather than purely technical procurement criteria. Future procurement and system configuration processes should include formal validation of DICOM workflow integrity, worklist enforcement, metadata handling, and exception recovery (1, 57, 58). In line with ISO 14971, preventive controls should prioritize design-based safeguards and protective system controls over user-dependent procedural controls (23, 36).

These recommendations should not be implemented as isolated interventions. From a sociotechnical systems perspective, improvements in workflow governance, metadata validation, interoperability, system configuration, and user practices are interdependent, and changes in one component may influence the performance of others (6, 8, 53, 59–61). Consequently, effective risk reduction requires joint optimization of technical, organizational, and human factors rather than independent modification of individual system components. Implementing these interventions as an integrated strategy is therefore more likely to improve workflow resilience and reduce unintended consequences than introducing isolated corrective actions.

Taken together, these measures shift incident management from reactive correction to proactive workflow resilience. The risk–control matrix identified system-level controls that could be targeted to reduce recurrence, including booking enforcement, worklist validation, identity control, visible system alerts, and exception handling. However, because these controls were not implemented or evaluated within the case, their effect on residual risk could not be quantified. The resulting preventive and corrective strategies are summarised in Box 2.

BOX 2 **Preventive and corrective strategies for preventing recurrence of workflow-integrity and interoperability failures in ultrasound imaging**

**Workflow governance and examination initiation**

- Enforce mandatory booking completion before examination initiation to ensure that each study is associated with valid scheduling and accession metadata.
- Require modality worklist use as the default pathway for all routine examinations and restrict examination initiation when no valid worklist entry is available.
- Establish structured exception workflows for urgent examinations, including temporary emergency identifiers and controlled *post hoc* reconciliation.
- Prevent examination completion when essential identity fields or accession-linked metadata are incomplete, invalid, or absent.

**Identity integrity and metadata control**

- Restrict manual patient identity entry to formally defined exception scenarios and require secondary verification before image acquisition.
- Implement automated validation of patient identifiers, accession numbers, and study metadata at the point of entry and before image transfer.
- Introduce visible system warnings when worklist linkage is absent, patient identity fields are incomplete, or manually entered identifiers do not match expected formats.
- Standardise identity verification procedures at the modality to ensure consistent confirmation of patient identity before acquisition and transfer.

**System safeguards and interoperability resilience**

- Configure ultrasound, storage, and downstream archive systems to reject or quarantine studies with invalid, incomplete, or unreconciled metadata rather than allowing silent progression.
- Introduce automated exception handling for unreconciled studies, including quarantine queues, escalation alerts, and controlled recovery pathways.
- Ensure that downstream archive systems can detect, flag, and reconcile invalid or incomplete studies before they become inaccessible in routine retrieval.
- Strengthen interoperability validation across booking, modality worklist, acquisition, and storage systems to ensure metadata continuity across the full imaging pathway.

**Training, monitoring, and organisational control**

- Train sonographers and imaging staff in the relationship between booking logic, worklist population, metadata integrity, and downstream image availability.
- Provide periodic refresher training on exception workflows, urgent examinations, identity handling, and manual override procedures.
- Monitor workflow deviations prospectively, including missing bookings, worklist bypass, manual identity entry, and delayed study reconciliation, as routine safety indicators.
- Establish local escalation and audit mechanisms for repeated workflow bypass, metadata validation failure, and unresolved, unverified studies.

### Implications for digital imaging safety

This case highlights a broader safety challenge in digital imaging: clinically significant failures may arise not from image-quality degradation but from failures of the digital conditions required to identify, store, retrieve, and trust imaging information. In such cases, patient risk arises from information inaccessibility, identity ambiguity, and reduced traceability rather than from direct diagnostic inaccuracy. This distinction is increasingly important in digitally dependent imaging environments, where safe clinical care depends as much on metadata integrity and workflow interoperability as on image acquisition itself (2, 62–64).

This case also suggests that the risk of patient misidentification in imaging may extend beyond acquiring images of the wrong patient. Misidentification may also occur downstream when valid images become associated with incomplete, incorrect, or unverifiable identity data. This broadens the conventional understanding of imaging-related identification risk and highlights the need to treat metadata integrity as a patient safety priority rather than a purely technical concern.

This case's contribution extends beyond applying multiple analytical frameworks. Previous studies have demonstrated the value of combining complementary approaches to examine health information technology–related safety events; however, this case applies that integrated perspective to a specific ultrasound imaging workflow in which the primary failure involved the continuity of booking, patient identity, worklist, and downstream metadata. The case therefore illustrates a distinct form of imaging safety risk in which image acquisition can be technically successful while the associated examination information becomes incomplete, unverifiable, or difficult to retrieve (20, 29, 65, 66). In particular, the case shows how metadata integrity and identity continuity can become patient-safety concerns downstream of acquisition, thereby extending the interpretation of imaging interoperability beyond technical data exchange to the preservation of reliable clinical traceability across the imaging workflow (6–8, 19, 20).

### Strengths and limitations

A major strength of this study is the use of multiple complementary analytical frameworks to examine the same incident from patient safety, technical, workflow, and risk-control perspectives. This strengthened interpretive validity through methodological triangulation and reduced reliance on single-framework interpretation (49). The use of a validated 18-step workflow model also improved methodological rigor by anchoring workflow localization within an established imaging framework rather than relying on case-specific retrospective reconstruction (17).

A second strength is the integration of descriptive incident classification with prospective risk-informed analysis (67). Rather than limiting the case to retrospective description, the study translated incident findings into practical preventive and corrective controls relevant to digital imaging safety and clinical workflow resilience (68, 69).

This study has limitations. First, the analysis was based on a single incident case and cannot establish prevalence or causal generalisability. Second, the case was derived from an anonymized, voluntary incident-reporting repository and therefore depended on the completeness and accuracy of the original report and the internal investigation. As with all incident-report–based analyses, under-reporting and reporting bias remain possible. Third, the study relied on secondary analysis of existing documentation and did not include direct system observation, user interviews, or prospective workflow testing. Nevertheless, the case remains analytically valuable because it demonstrates how recurrent low-visibility workflow deviations can propagate into clinically significant HIT-related patient safety risks.

In addition, the mini-root cause analysis was based on retrospective incident documentation rather than direct observation or prospective investigation. Consequently, the analysis may not have captured undocumented contextual factors, organizational decision-making processes, or informal workflow adaptations that influenced the incident. Nevertheless, the use of multiple complementary analytical frameworks strengthened the credibility of the findings by enabling triangulation across technical, workflow, patient safety, and risk management perspectives (33, 34).

As a retrospective single-case study, the findings should be interpreted as providing analytical rather than statistical generalizability. Although the specific workflow configuration, organizational context, and technical environment may differ across healthcare organizations, the methodological approach presented in this study may be transferable to investigating similar health information technology–related incidents in comparable digital imaging settings (17, 18, 70, 71). The value of this study, therefore, lies in demonstrating the application of a structured multi-framework analytical approach for understanding complex sociotechnical failures and informing system-level improvement, rather than in claiming that the observed findings are universally applicable (33, 34).

## Conclusion

This case demonstrates that technically successful ultrasound image acquisition can coexist with failures in the digital workflow required for reliable patient identification, storage, retrieval, and interpretation of imaging information. The incident originated with an upstream booking failure and propagated through workflow bypass and identity/metadata problems, resulting in unverified examinations and delayed or difficult retrieval. No confirmed patient harm occurred, but the observed failures created potential patient-safety and information-governance hazards.

The case extends previous multi-framework analyses by demonstrating how this integrated approach can be applied to a specific ultrasound interoperability pathway in which metadata and identity continuity become critical determinants of imaging safety after acquisition. The findings highlight that imaging interoperability should be understood not only as successful technical data exchange, but also as preservation of accurate identity, workflow linkage, traceability, and retrievability across the imaging chain.

## Data Availability

The original contributions presented in the study are included in the article/Supplementary Material, further inquiries can be directed to the corresponding author.
