# Supplementary material for: Patient misidentification and data integrity risks in ultrasound imaging: a case report using multi-framework incident analysis
Source: Front Health Serv. 2026 Sep 3;6:1872556. doi: 10.3389/frhs.2026.1872556 (PMC13582320; doi:10.3389/frhs.2026.1872556)
Supplement: Supplementary file 1 [file Table1.docx]

**Table 4. Risk–control matrix linking workflow hazards, contributing factors, existing safeguards, proposed preventive and corrective controls, and residual risk across the ultrasound imaging workflow.**

| **Process step** | **Failure mode** | **Contributing cause** | **Potential consequence** | **Initial risk** | **Existing safeguards** | **Proposed preventive controls** | **Proposed corrective controls** | **Residual risk assessment** |
| --- | --- | --- | --- | --- | --- | --- | --- | --- |
| Appointment booking | No booking created | Lack of knowledge, emergency workflow, time pressure | No valid accession-linked workflow information; examination remains in downstream workflow; risk of data loss | High | Post-event booking creation; manual MTA matching | Mandatory emergency booking template; booking validation before examination | Post-event booking and manual reconciliation where required | Not formally calculated; post-control reassessment required |
| Appointment booking | Incorrect examination type selected | Cognitive error; unclear booking options | Examination may not route correctly | Medium | Manual recoding and reconciliation | Standardized examination codes; restricted free-text options | Manual recoding where required | Not formally calculated; post-control reassessment required |
| Modality worklist | DICOM worklist not used | User bypass; workflow awareness | Examination cannot be reliably archived | High | Manual correction where identifiable | Mandatory worklist selection; system enforcement | Manual reconciliation of affected studies | Not formally calculated; post-control reassessment required |
| Patient identity handling | Manual identity entry | Emergency workflow; user workaround | Invalid or corrupted patient identifier | High | Manual review/correction; examination quarantine where applicable | Restrict manual identity entry; identity validation | Quarantine and reconciliation of affected examination | Not formally calculated; post-control reassessment required |
| Patient identity handling | Wrong patient selected | Identity-check failure; workflow pressure | Incorrect attribution; potential patient-safety risk | Very high | Incident reporting; repeat examination where necessary | Two-step identity verification; confirmation prompts | Patient-safety investigation and examination repeat where required | Not formally calculated; post-control reassessment required |
